# Notes and News

**Published:** 1904-09-24

**Authors:** 


### Notes and News.

The inquiry into the complaints against the tinned meats supplied to the *Discovery* will be looked for with interest. Health at sea, whether in relation to scientific research, commerce, or the Navy, is so dependent upon this source of supply that it is of the utmost importance that such supervision over stores should be exercised as to render inferior or unwholesome supplies impossible.

A man named John Tucker has been practising as a medical man in this country, and recently in partnership with a Dr. Gould in Liverpool. Dr. Gould has been absent in South Africa for some time, and had dissolved the partnership. Tucker carried on the practice and signed death certificates in Dr. Gould's name, for which he has been convicted of forgery. Tucker is believed to possess a Pennsylvanian M.D. degree.

The City Orthopædic Hospital is keeping up a protest before the public, against the Hospital Sunday Fund, which has this year withheld a grant to it. The hospital authorities must know very well that public opinion is frequently formed without any investigation into the facts at issue, and it is easy enough to trade on a want of technical knowledge to arouse a sentimental sympathy. Cruelty to the sick poor of Hatton Garden is pleaded, because they are asked to locate themselves elsewhere in better quarters. How few of the public realise that the patients attending an orthopædic hospital spend a far longer and drearier time in bed, or in the ward, than is usual for patients in a general hospital, hence the necessity for the best surroundings. Also that the cases are seldom in urgent need of immediate treatment, so that it is quite convenient to remove the patients to a place where their recovery is likely to be expedited. Visitors to Hatton Garden, even if they are not experts in matters of hygiene, we feel sure will agree that there are other than economic advantages in removing the in-patients elsewhere. Surely arrangements could be made for the out-patients to be treated locally, without interfering with the amalgamation scheme, which from the in-patients' point of view has so much to recommend it.

In spite of the small-pox epidemic at Dewsbury, the football match has been held, and also a local fête. At the moment the number of cases shows a decrease.

A dairyman in New Orleans has had the effrontery to sue the City Health Officer and issue a threat to the City Board of Health because milk was seized coming from his dairy, it being ascertained that there were two cases of typhoid fever on his premises, where the dairy arrangements were very unsanitary.

An account has appeared in the *Daily Mail* of a Servian man aged 117 years. The account of his habits will be bitter reading to teetotallers. It is said that in his youth he drank about 12 litres of wine a day, and now in his extreme old age, which he bears so lightly, he consumes about three-quarters of a litre of brandy daily. He commences the day at seven with a glass of this spirit. He has never smoked or performed regular work. The popular truism that "a man is as old as his arteries" has application here. Statistics presented at a recent meeting of the American Medical Association bore testimony to the fact that hard physical labour is undoubtedly a very important factor in inducing precocious senility, due to degeneration of the arteries. The example of Zikitsch is not likely to be quoted by the advocates of industry and temperance.

Mr. Stephen Coleridge, of the Antivivisection Society, is again engaged in attempting to injure the cause of the great general hospitals with medical schools in London, and to deprive them of the support of the giving public, to which they undoubtedly have the first claim. He has gone into statistics which, to use his own words, "led to the startling discovery that the sick poor, so far from benefiting by the disseminated knowledge of the schools and the superior dexterity of the vivisectors, ran a far greater risk of dying in these particular hospitals than in the others unconnected with schools licensed for vivisection." He has drawn up a return, in which a comparison is made between the death-rate at the hospitals with medical schools and that at the other hospitals in London. It is well known that the cases at general hospitals are for the most part of a serious nature and made up of acute cases and bad accidents, while the second list of hospitals is a miscellaneous one, and includes institutions for the reception of chronic diseases and cottage hospitals where minor cases are received. It will therefore be obvious that the work of these two groups of institutions does not admit of comparison. (We wonder why Mr. Coleridge left the Poplar Hospital out of his miscellaneous group. That hospital is engaged in a splendid work, but unfortunately, owing to the serious nature of its cases, its figures would tend to upset Mr. Coleridge's comparison.) However, for the purposes of a fair comparison, we have taken the trouble to pick out from the miscellaneous group the institutions which are engaged in general hospital work, and we find that the death-rate per bed is virtually the same as at the general hospitals with medical schools—viz. 1·18. This is, of course, what would naturally be expected.

